# Staphylococcal trafficking and infection—from ‘nose to gut’ and back

**DOI:** 10.1093/femsre/fuab041

**Published:** 2021-07-14

**Authors:** Elisa J M Raineri, Dania Altulea, Jan Maarten van Dijl

**Affiliations:** Department of Medical Microbiology, University of Groningen, University Medical Center Groningen, Groningen, PO Box 30001, 9700 RB, the Netherlands; Department of Medical Microbiology, University of Groningen, University Medical Center Groningen, Groningen, PO Box 30001, 9700 RB, the Netherlands; Department of Medical Microbiology, University of Groningen, University Medical Center Groningen, Groningen, PO Box 30001, 9700 RB, the Netherlands

**Keywords:** reservoir, colonization, infection, gut, nasopharynx, immune cells

## Abstract

*Staphylococcus aureus* is an opportunistic human pathogen, which is a leading cause of infections worldwide. The challenge in treating *S. aureus* infection is linked to the development of multidrug-resistant strains and the mechanisms employed by this pathogen to evade the human immune defenses. In addition, *S. aureus* can hide asymptomatically in particular ‘protective’ niches of the human body for prolonged periods of time. In the present review, we highlight recently gained insights in the role of the human gut as an endogenous *S. aureus* reservoir next to the nasopharynx and oral cavity. In addition, we address the contribution of these ecological niches to staphylococcal transmission, including the roles of particular triggers as modulators of the bacterial dissemination. In this context, we present recent advances concerning the interactions between *S. aureus* and immune cells to understand their possible roles as vehicles of dissemination from the gut to other body sites. Lastly, we discuss the factors that contribute to the switch from colonization to infection. Altogether, we conclude that an important key to uncovering the pathogenesis of *S. aureus* infection lies hidden in the endogenous staphylococcal reservoirs, the trafficking of this bacterium through the human body and the subsequent immune responses.

## Teaser

Here, we review the roles of different reservoirs in the human body in the onset of infections caused by the pathogen *Staphylococcus aureus* with focus on mechanisms of bacterial trafficking and the switch between colonization and infection.

## INTRODUCTION

*Staphylococcus aureus* is an opportunistic human pathogen that is infamous for causing community- and hospital-acquired infections. When *S. aureus* unfolds its pathogenic nature, it can cause many pathologies, including infections of the skin, wounds, soft tissues, bloodstream, bones and lungs. In addition, the contamination of food products with *S. aureus* may lead to serious cases of gastroenteritis. In recent years, *S. aureus* has become the leading cause of bloodstream infections (Thwaites and Gant 2011; Guimaraes *et al*. 2019; Turner *et al*. 2019). The treatment of such staphylococcal infections is, unfortunately, becoming increasingly difficult due to the emergence of multiple drug resistance, which is best exemplified by the methicillin-resistant *S. aureus* (MRSA) lineages (Corey 2009; Thwaites and Gant 2011). Once *S. aureus* is in the bloodstream, it can reach the different tissues and organs of the human body, thereby causing metastatic infections. Due to its resistance to most clinically approved antibiotics, treatment of *S. aureus* infections and eradication of this pathogen from the human body is often incomplete, leading to recurrent infections (Foster 2017). However, the persistence of *S. aureus* in the body is related to not only drug resistance but also effective mechanisms employed by the pathogen to evade the human immune defenses and its ability to hide in particular ‘protective’ niches (Kubica *et al*. 2008; Thwaites and Gant 2011; Horn *et al*. 2017; Mekonnen *et al*.^2017^, ^2018^). This is remarkably underscored by the fact that *S. aureus* is capable of surviving inside immune cells like monocytes, macrophages and granulocytes, and even in dendritic cells (Horn *et al*. 2017; Balraadjsing *et al*. 2019).

Due to its high adaptability to different environmental conditions, the opportunist *S. aureus* has become an integral part of the human microbiome, where it can persist asymptomatically for prolonged periods of time. Here, one has to differentiate between persistent carriers, who are always colonized by *S. aureus*, and intermittent carriers, who present *S. aureus* with varying frequency (Wertheim *et al*. 2005; Mulcahy and McLoughlin 2016; van Belkum 2016). However, the difference between persistent and intermediate carriage is vague, because *S. aureus* may be hiding at body sites that are not sampled at the time of examination. For instance, in most studies, samples are taken from the anterior nares or the skin, whereas the perineum and gastrointestinal (GI) tract are less frequently sampled sites where *S. aureus* often resides (Acton *et al*. 2009; Sakr *et al*. 2018). In particular, intestinal carriage can occur in the absence of nasal carriage, whereas nasal carriage has been associated with increased *S. aureus* intestinal carriage (Acton *et al*. 2009). The noncarriers are the remaining part of the population, representing a minority of people where *S. aureus* is hardly ever detectable. The latter however does not rule out the possible existence of hidden reservoirs. Also, the noncarriers usually show significant antistaphylococcal immunoglobulin levels, suggesting that they have a history of contacts with the pathogen, including incidental contaminations and perhaps minor infections that passed unnoticed (Verkaik *et al*. 2009; Sollid *et al*. 2014).

The skin and the mucosa of the human body are usually regarded as physical barriers against external insults, but they actually represent networks of effector cells and molecular mediators that constitute a complex immune system. Once these protective barriers of the human body are breached, for instance by trauma, surgery or viral infections, the underlying body layers are exposed, granting easy and rapid access for pathogens like *S. aureus* to deep-seated tissues and the bloodstream (Abdallah, Mijouin and Pichon 2017). This opens the gate for dissemination of *S. aureus* throughout the body with serious health hazards. For example, the epithelial cell layer of the human lung forms an important primary barrier against infection. However, upon a breach of this barrier, or during the early stages of tissue regeneration, the options to mount effective responses to the staphylococcal insult are inadequate (Palma Medina *et al*. 2020). Likewise, the dynamics of *S. aureus* infection of endothelial cells was shown to be highly dependent on the integrity of the endothelial barrier (Raineri *et al*. 2020). In recent years, the numbers of surgical interventions in different parts of the human body have steeply increased due to aging of the population, with MRSA being one of the most frequently encountered causative agents of surgical site infections (Fukuda *et al*. 2020).

Once the epithelial or endothelial barriers have been breached, the innate and adaptive immune defenses impose the main barriers against invasive staphylococcal infections of deeper seated tissues and the bloodstream. The interaction between the immune system and *S. aureus* can go in two directions. In one scenario, the bacteria are effectively killed by the complement system or phagocytic immune cells, leading to the prevention of infectious disease. Alternatively, the bacteria manage to evade the immune defenses, either by killing of phagocytes, intraphagocyte survival, intracellular persistence (within the cytoplasm or organelles) or biofilm formation, which will lead to asymptomatic colonization of the host, chronic infection or fulminant pathology (Voyich *et al*. 2005; Bhalla, Aron and Donskey 2007; Thurlow *et al*. 2011; Flannagan, Heit and Heinrichs 2015; Thammavongsa *et al*. 2015; Lubkin and Torres 2017; Darisipudi *et al*. 2018). Throughout its evolution, *S. aureus* has acquired a plethora of factors that allow this pathogen to evade, manipulate and subvert the host immune defenses, making it one of the most successful pathogens ever (Thammavongsa *et al*. 2015).

**Figure 1.**
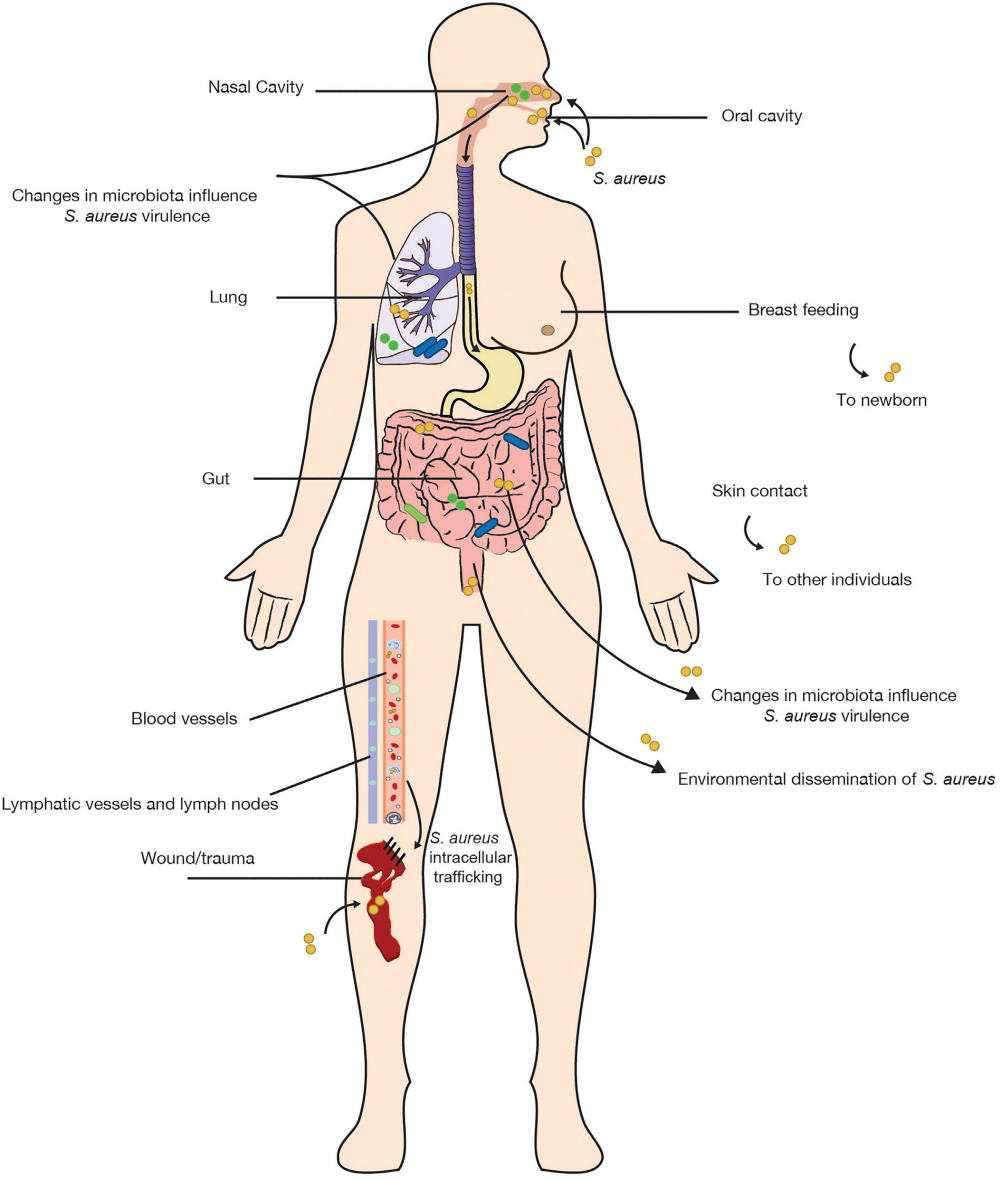
Routes of *S. aureus* acquisition, dissemination in the human body and transmission. *Staphylococcus aureus* can enter the human body via direct or indirect interpersonal contacts, contaminated food products, trauma and surgery. Following contamination and colonization, *S. aureus* may be disseminated to different body sites. As a consequence, *S. aureus* may reside in the nasal cavity, oral cavity, gut and lungs, or on the skin. Translocation of *S. aureus* between these different sites may relate to changes in the complexity of the nasal, oral, gut, lung or skin microbiota, infectious diseases, trauma or surgery. Immune cells in the mucosa, in tissues, and in the vasculature and lymphatic system can contribute to the staphylococcal dissemination within the body. Transmission of *S. aureus* to newborns may take place through breastfeeding and parental skin contact. Lastly, the bacterium can be disseminated from the gut into the environment, which may lead to its transmission to other individuals via the fecal–oral route. Arrows indicate directions of bacterial dissemination, and solid lines mark relevant anatomical sites.

Upon contact with the human host, the bacterial cells need to establish firm interactions with cell surfaces, tissues or implanted devices, in order to colonize the host for extended periods of time (Sakr *et al*. 2018). Over the past decades, much research has been focused on *S. aureus* colonization of the most common endogenous niches, especially the nasopharynx and oral cavity, while the frequency of intestinal colonization has remained relatively underestimated. The aim of this review is to focus attention on the endogenous reservoirs of *S. aureus* in the human host. We highlight recently gained insights in the role of the human gut as an endogenous *S. aureus* reservoir next to the more intensely investigated nasopharyngeal and oral *S. aureus* reservoirs. From its different ecological niches, the pathogen can disseminate to other parts of our body as schematically represented in Fig. 1. In this context, we address the interactions of *S. aureus* with different types of blood cells as possible vehicles for staphylococcal dissemination.

## THE HUMAN NASOPHARYNX AND ORAL CAVITY

**Figure 2.**
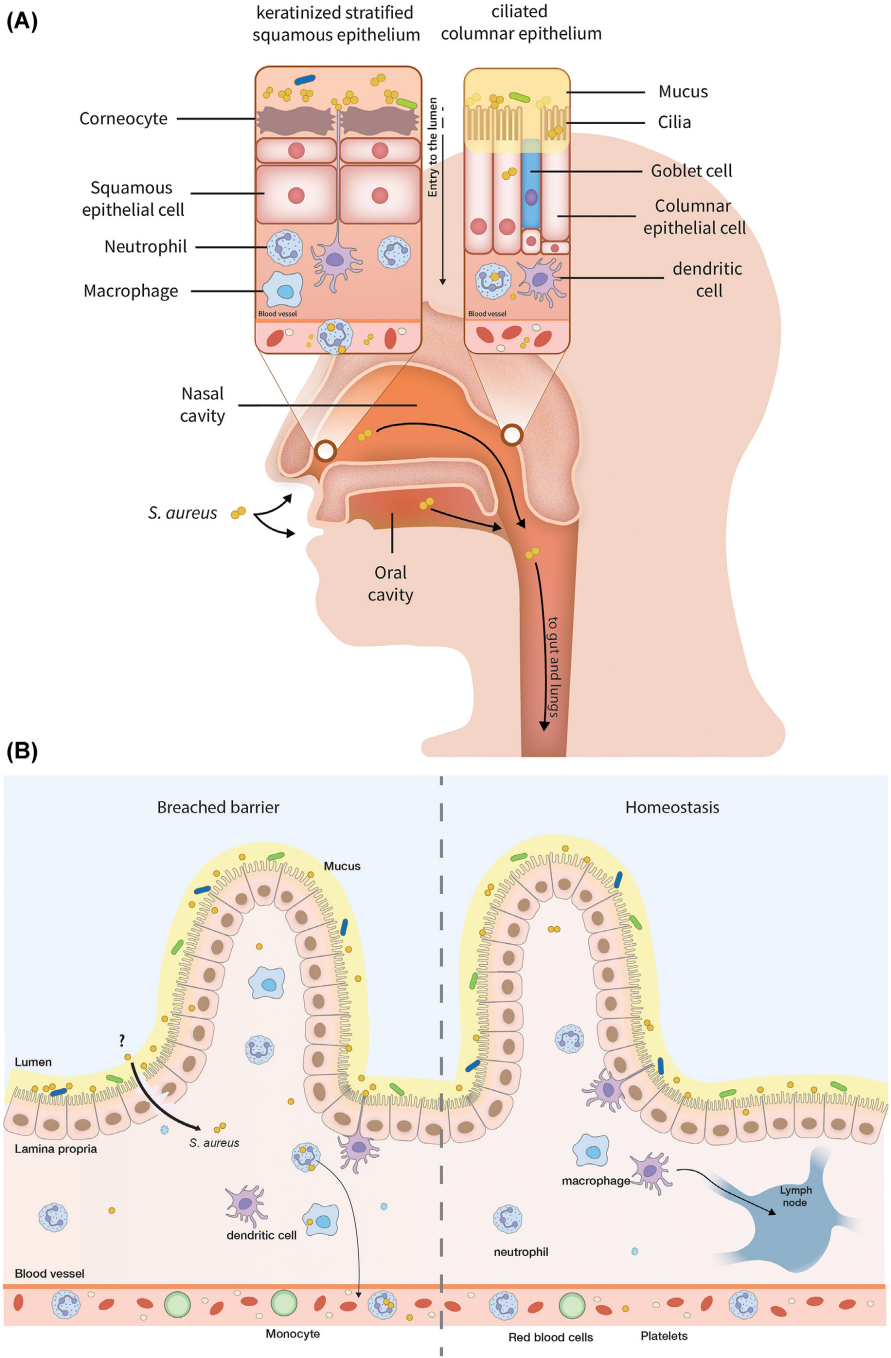
A proposed model for *S. aureus* colonization of the nasopharynx and the human gut, and mechanisms that promote bacterial dissemination to various parts of the human body. **(A)***Staphylococcus aureus* frequently resides both in the nasal and oral cavities. In the nasopharynx, *S. aureus* interacts with different cells of the epithelium, the mucus layer, coresident nasal microbiota and immune cells. These interactions and factors, such as active disruption of the nasal barrier by other microorganisms, host-immune failure and inflammation, may help *S. aureus* to translocate into deeper seated tissues, cavities and blood vessels, and from there to other body sites.**(B)** Following ingestion, surgery or translocation from the bloodstream or lymphatic system, *S. aureus* may reach the gut. Upon gut colonization, *S. aureus* interacts with the mucus layer, different cells of the intestinal epithelium, coresident gut microbiota and immune cells. These interactions and factors, such as active disruption of the gut barrier by other microorganisms, host-immune failure, changes in the gut permeability due to inflammation and gut health (e.g. dysbiosis), may help *S. aureus* to translocate from the mucus layer into deeper seated tissues and blood vessels. However, the mechanisms that allow *S. aureus* to colonize the human gut or to breach the human gut barrier need to be further investigated.

The nasal cavity is a complex structure of the human body where several bacteria reside, and the composition of its microbiota changes with function of time and the human host characteristics. This compartment is lined by a keratinized stratified squamous epithelium in the anterior part and by a columnar ciliated epithelium in the inner part (Fig. 2A) (Weidenmaier 2012). *Staphylococcus aureus* persistently colonizes the nasopharynx of approximately one-fifth of the human population. A higher rate of nasal colonization is found in children, amounting to around 45% in the first weeks of life. However, *S. aureus* nasal carriage decreases with time (Wertheim *et al*. 2005). Furthermore, the nasal carriage rate is determined by sex, ethnicity, age, history of disease and the immunity of the human host (Liu *et al*. 2015; Sakr *et al*. 2018). Host genetic determinants were shown to be important for *S. aureus* nasal colonization, but the microbiota also influences *S. aureus* abundance in the nasopharynx (Liu *et al*. 2015). In case of persistent colonization, it was observed that *S. aureus* can show a niche adaptation to the host environment, but the presence of single-nucleotide polymorphisms (SNPs) and of genetic variations in the host genome may influence the colonization outcome (Mulcahy and McLoughlin 2016). For example, such variations were detected in genes encoding IL-4, C-reactive proteins, Toll-like receptors (TLR), mannose-binding lectin and the DEFB1 defensin of persistent *S. aureus* nasal carriers (Mulcahy and McLoughlin 2016; Shepherd and McLaren 2020). Furthermore, the *S. aureus* strains isolated from nasal human carriers were shown to have an effect on the local immune response in the nose. For example, this was observed in nasal epithelial cells, where the human β-defensin was downregulated, or where the upregulation of TLR-2 was delayed (Quinn and Cole 2007). Next to the nasal cavity, the oral cavity and perioral regions are also important niches from where *S. aureus* can disseminate to other body sites and take part in certain oral diseases. This view was underscored by screening for MRSA and methicillin-sensitive *S. aureus* (MSSA) in the oral cavity, which allowed the detection of strains that would have been overlooked by only sampling the nasal cavity (McCormack *et al*. 2015; Kearney *et al*. 2020).

The transmission of bacteria residing in the nasal and oral cavities mainly occurs via direct or indirect interpersonal contacts (e.g. mother–infant contacts or contacts with health care workers) and is facilitated by nose picking, airborne saliva droplets and contaminated surfaces (Wertheim *et al*. 2004; Sakr *et al*. 2018). The resulting colonization of the nasal mucosa could, in many instances, be linked with the development of *S. aureus* bacteremia (von Eiff and Peters 2001; Wertheim *et al*. 2005; Sakr *et al*. 2018). For example, 15% of a cohort of preclinical medical students in Nepal displayed nasal *S. aureus* colonization and, importantly, screening for nasal colonization helped to decrease the transmission of *S. aureus* from community to hospital settings (Ansari *et al*. 2016). During the early steps of colonization, *S. aureus* adhesins will establish interactions with host cell molecules of the skin and mucosa. For instance, this can occur via the binding of the cell wall-anchored ClfB and IsdA proteins to the cornified envelope of the stratum corneum, or via cell wall-anchored proteins with the host cell receptor SREC-I that is present on the surface of ciliated epithelial cells (Weidenmaier 2012; Leonard, Petrie and Cox 2019). When colonizing the nasopharynx, *S. aureus* will interact both with the squamous epithelium and with the cells of the ciliated columnar epithelium in the inner nasal cavity (Fig. 2A). Additionally, *S. aureus* adheres to the mucosa in the nasopharynx via adhesin-receptor interactions of bacterial proteins and the carbohydrate moiety in the mucin (Shuter, Hatcher and Lowy 1996).

Next to the extracellular location of colonizing *S. aureus*, an intracellular localization of this bacterium has been observed in both epithelial and endothelial cells, and even in inflammatory cells such as mast cells (Ou *et al*. 2016; Sakr *et al*. 2018). Importantly, the intracellular survival of *S. aureus* in cells of the nasal cavity, including the nasal epithelium (Fig. 2A), glandular cells and myofibroblastic cells, was shown to be a determinant for recurrent infections in patients with *S. aureus* rhinosinusitis (Clement *et al*. 2005). Such findings suggest an important role for the nasal mucosa as a silent intracellular reservoir for bacterial survival leading to recurrent infections (Clement *et al*. 2005; Jeon *et al*. 2020). Development of infection from endogenous sources is also believed to occur in ventricular assist device infections. In fact, the endogenous presence of *S. aureus* in the nasopharynx, was shown to be a risk factor for ventricular assist device infection, which usually occurs from 7 weeks to 1 year after the implantation (Nurjadi *et al*. 2020). These evidences of intracellular survival of *S. aureus* inside nonphagocytic cells of the nasal tissues and of intracellular survival in phagocytic cells could be a starting point for so-called silent intracellular trafficking of *S. aureus* from the nasal cavity to other body sites and the onset of infection. This will depend on factors that diminish the nasal barrier homeostasis over time and influence the entry of immune cells that can serve as carriers of the intracellular *S. aureus*.

## THE HUMAN GUT

The human gut is an organ that serves multiple functions in the absorption of water, the digestion and uptake of nutrients, and in shaping the immune system. These functions are supported by a plethora of different gut-resident microorganisms that actually outnumber the total number of human cells >10-fold (Thursby and Juge 2017). In a healthy individual, the intestinal barriers provide the body with an effective defense line against environmental factors and the gut-resident microbiota, which includes many different opportunistic pathogens (Kamada *et al*. 2013). At the same time, the intestinal barriers allow important crosstalk between the gut microbiota and the immune system (Takiishi, Fenero and Câmara 2017). The human GI tract is covered by a mucus layer, which maintains a homeostatic relationship with our gut microbiota and prevents the translocation of microbes to the underlying tissues (Turner 2009). Intestinal mucus is made of a glycoprotein network with a host-specific glycan structure, which, if disrupted, allows bacterial invasion of the epithelium causing inflammation and infection (Schroeder 2019). *Staphylococcus aureus* interacts with the mucus layer and this layer seems to be required to establish intestinal colonization. In fact, it has been shown that cecal mucus facilitates colonization of the intestinal tract by MRSA in a murine model (Gries, Pultz and Donskey 2005). Below the mucus layer there is the intestinal epithelium, which is composed of a single layer of multiple cells and junctions that separate the gut lumen from the underlying lamina propria. The lamina propria contains immune cells, including dendritic cells, macrophages and neutrophils. In particular, the dendritic cells and neutrophils may travel to underlying blood and lymph vessels (Takiishi, Fenero and Câmara 2017). Due to this structure of the gut, there is a close connection between different parts of the gut, blood vessels and more distant body sites, where immune cells may not only serve as messengers of signals and guardians against infectious agents, but also as ‘trojan horses’ that give *S. aureus* access to otherwise well-guarded body sites (Fig. 2B) (Suzuki 2013).

The GI tract is colonized by a large number of microorganisms, including bacteria and fungi. These microbes will start to colonize the human GI tract immediately after birth. During adulthood the complexity of the microbiota in the GI tract increases, and the respective microorganisms evolve different interactions between each other and with the human host. In recent years it has become increasingly clear that *S. aureus* is a common bowel colonizer in infants, which may affect the host's immune system (Acton *et al*. 2009). However, also in healthy adults and hospitalized patients *S. aureus* is a regular resident of the gut (Benito *et al*. 2015; Claassen-Weitz *et al*. 2016; Dong *et al*. 2018; Ray *et al*.^2003^). The frequency of carriage in healthy individuals and patients is ∼20% on average, but the actual carriage numbers may vary depending on the health condition and age. In particular, the human host responses play decisive roles in the outcome of colonization, and the intestinal colonization by *S. aureus* is therefore considered as an important risk factor for infection, as is the case for all intestinal pathogens (Gagnaire *et al*. 2017; Pickard *et al*. 2017; Dong *et al*. 2018, 2019).

From the gut, *S. aureus* can in principle reach other body sites through translocation across the mucosa and epithelium. This may relate to increased intestinal permeability caused by regular epithelial regeneration, diminished gut health due to inflammatory disorders or infection, or surgery. Alternatively, *S. aureus* translocation may follow active damage of the epithelium through the secretion of inflammatory compounds, allergens or toxic products (Lee, Moon and Kim 2018). This view is supported by *in vitro* experiments showing that *S. aureus* α-toxin can perturb the barrier function in Caco-2 epithelial cell monolayers by altering the junctions between the cells (Kwak *et al*. 2012). In addition, the bacterial translocation may be facilitated by changes in the intestinal microbiota and a host immune failure. For example, in patients with intestinal bowel disease (IBD), there is an increased intestinal permeability that triggers a cascade of events resulting in increased bacterial growth and risk of sepsis (Kumar *et al*. 2020). Notably, it was shown that staphylococcal superantigens are not causing the lesions of IBD, but *S. aureus* infection can occur during the time course of IBD (Chiba *et al*. 2001). Alternatively, gut-resident *S. aureus* may be (self-)transmitted to the perineum, skin, mouth or nasopharynx of the carrier and subsequently cause infections. Likewise, intestinal colonization will contribute to environmental contamination and staphylococcal dissemination (Acton *et al*. 2009). In turn, this can lead to fecal–oral transmission to other individuals via contaminated drinking water or food (Kadariya, Smith and Thapaliya 2014). Moreover, intestinal *S. aureus* colonization may enhance community- and nosocomial transmission and represents a serious risk factor for infections (Vesterlund *et al*. 2006; Bhalla, Aron and Donskey 2007; van Belkum 2016; Gagnaire *et al*. 2017, 2019). For instance, it was shown that diarrheal stools of patients colonized with MRSA have an important impact on the environmental contamination with these multiple drug-resistant variants, and it has even been evidenced that the intestinal tract could provide a potential reservoir for the much feared emergence of vancomycin-resistant *S. aureus* (Claassen-Weitz *et al*. 2016).

In early life, *S. aureus* may employ different (indirect) pathways to translocate from and to the human gut. These include vertical mother-to-infant transmission, parental skin contact, breastfeeding with the use of immune cells as trojan horses, saliva and food (Lindberg *et al*. 2004; Thwaites and Gant 2011; Benito *et al*. 2015; Claassen-Weitz *et al*. 2016; Sakr *et al*. 2018). Early life has been shown to be an important period for the correct establishment of the gut microbiota and vertical mother-to-infant microbial transmission has an important role in the initial colonization of the neonatal gut. In fact, in the first year of a newborn's life, the gut microbiota dramatically changes through interactions with the developing immune system in the gut (Thursby and Juge 2017). Accordingly, *S. aureus* was shown to be common in the gut of infants (Lindberg *et al*. 2004, 2011; Nowrouzian *et al*. 2019). Additionally, the characterization of *S. aureus* strains isolated from feces of healthy neonates showed how breastfeeding can contribute to early *S. aureus* intestinal colonization, which may influence development of the immune system (Benito *et al*. 2015). In particular, it has been proposed that dendritic cells could be involved in the transfer of maternal bacterial strains to the infant gut through an entero-mammary pathway (Fig. 1) (Rodríguez 2014). Furthermore, a high rate of *S. aureus* colonization of the infant gut by flora from the parental skin was reported, which seems to relate to an inadequate competition with other gut-resident bacteria (Lindberg *et al*. 2011; Nowrouzian *et al*. 2019; Lindberg *et al*. 2004).

With adulthood the intestinal carriage of *S. aureus* decreases probably due to the increased complexity of the adult microbiota, which provides protection against colonization of the GI tract by exogenous microorganisms (Lindberg *et al*. 2004, 2011; Gagnaire *et al*. 2017; Dong *et al*. 2018). In this context it is noteworthy that the presence of endogenous lactic acid bacteria can decrease *S. aureus* colonization of the human intestinal mucus (Vesterlund *et al*. 2006). More recently, it was shown that the Gram-positive bacterial spore former *Bacillus subtilis* may contribute to the elimination of intestinal *S. aureus* through secretion of the lipopeptide fengycin, which interferes with the quorum-sensing that is fundamental to *S. aureus* colonization (Piewngam *et al*. 2018). Additionally, saliva and the binding of salivary proteins to *S. aureus* is thought to play an important role in preventing systemic infections (Heo *et al*. 2013). Nonetheless, it should be noted that *S. aureus* developed resistance to the antimicrobial activities of important saliva components, such as the human lysozyme and degradation products of this enzyme that function as cationic antimicrobial peptides, as exemplified by the LP9 peptide (Herbert *et al*. 2007). In addition, it has been shown in a murine model that *S. aureus* GI tract colonization can be modulated through the staphylococcal cell wall teichoic acid, capsule and surface proteins (Misawa *et al*. 2015). The latter observations provide insights into the various mechanisms that *S. aureus* employs to become an effective gut colonizer.

The endogenous carriage of *S. aureus* is a potential risk factor for frail hospitalized individuals. In particular, clinical studies have shown that carriage of MSSA or MRSA may lead to the development of community- or hospital-acquired infections in patients (Wolkewitz *et al*. 2011; de Kraker, Wolkewitz *et al*. 2011; de Kraker, Davey *et al*. 2011). In this context, *S. aureus* gut reservoirs appear to contribute substantially to the risk of infection and, in general, the endogenous reservoirs have important implications for hospital epidemiology (van Belkum 2016). A study on a long-term hospital outbreak of ST228 MRSA showed that it depended on asymptomatic intestinal carriage and on lack of identification of carriers over time (Senn *et al*. 2016). Furthermore, a meta-analysis of 712 studies has indicated that the intestinal carriage rate in healthy adults is ∼13.8% for *S. aureus* in general, and 1.4% for MRSA (Gagnaire *et al*. 2017). Although in healthy newborns, the carriage rate of *S. aureus* in general is ∼38.5% with 7.3% for MRSA, in children the carriage rate of *S. aureus* was shown to decrease to 23.4% with 3.1% for MRSA. The specimens considered in this study were from fecal, rectal, perineal and rectovaginal origin (Gagnaire *et al*. 2017). In another analysis, the intestinal *S. aureus* carriage in healthy Chinese individuals in the community was found to decrease with age, with the highest prevalence (6.15%) in youth, and the lowest (2.7%) prevalence in the elderly (Dong *et al*. 2018). The specimens considered in this study were only from fecal origin. A third systematic review investigating the presence of *S. aureus* in feces from hospitalized individuals and healthy individuals in the community, which involved different study population settings, estimated the overall carriage rate at 26% of which 86% was MSSA and 10% was MRSA (Claassen-Weitz *et al*. 2016). Lastly, a study on 363 ICU patients estimated the prevalence of *S. aureus* carriage from nasal samples (28%) and rectal samples (14%). Importantly, this study documented endogenous infection in patients with both rectal and nasal carriage, or with rectal *S. aureus* carriage only (Gagnaire *et al*. 2019).

**Figure 3.**
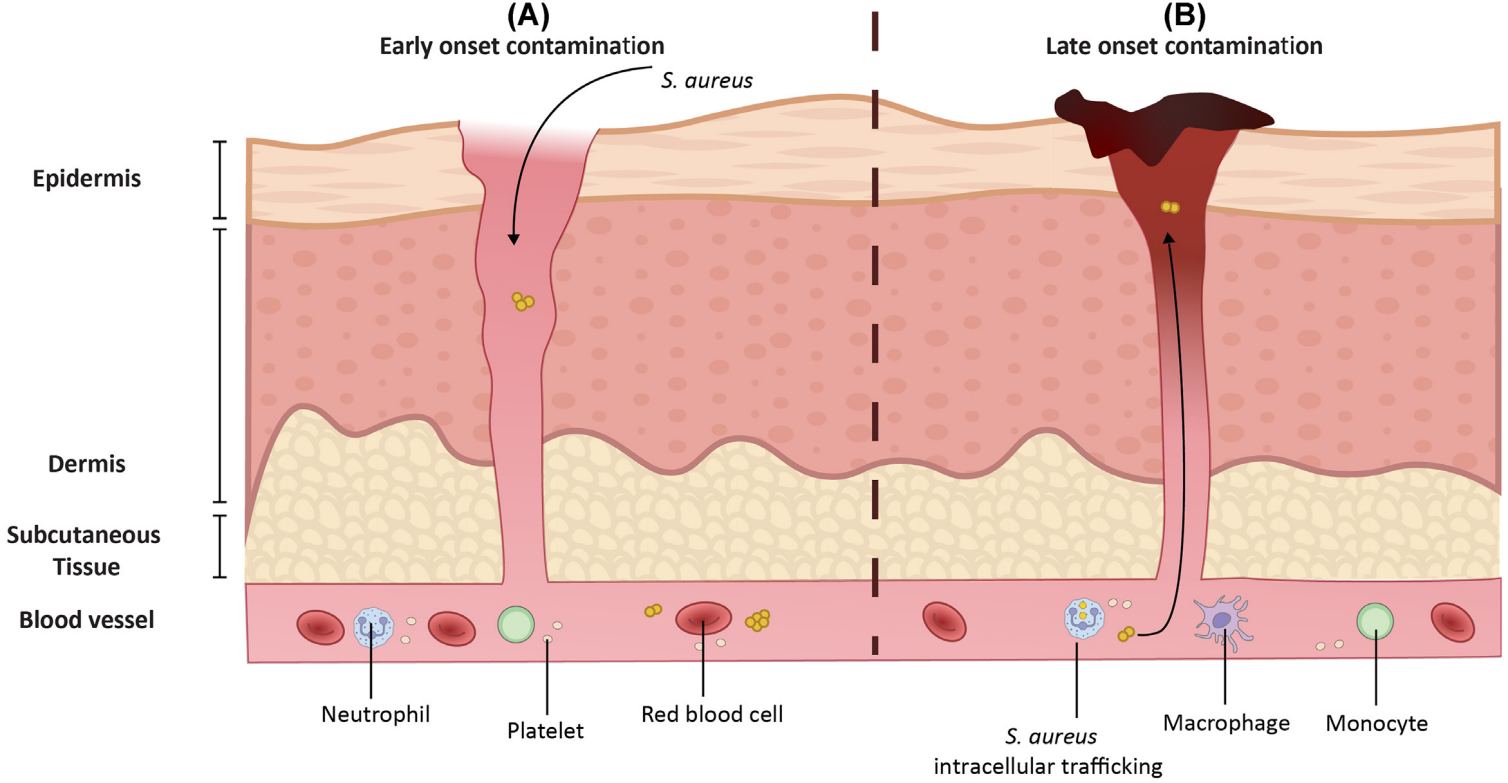
Schematic representation of postsurgical wound infection caused by *S. aureus*. **(A)** Early onset infections may be a consequence of wound contamination during surgery. Superficial surgical site infections affect the epidermis, dermis and subcutaneous tissue, but they may progress to deep-seated soft tissues and the bloodstream. **(B)** Surgical wounds may also be contaminated with *S. aureus* through a hematogenous route, which can explain late-onset infections after wound closure. In this case, the blood-borne *S. aureus* may originate from endogenous bacterial reservoirs in the nasopharynx, mouth, lungs or gut. Conceivably, this involves *S. aureus* hiding inside immune cells that are recruited to the surgical site and serve as Trojan horses.

In case bacteria reach the inner layers of the human GI tract, they will have to interact with cells of the immune system (Fig. 2B), which may involve endocytosis and subsequent destruction by phagocytes. However, the internalized *S. aureus* may survive inside professional phagocytes and dendritic cells, and the bacteria may even multiply intracellularly (Kubica *et al*. 2008; Horn *et al*. 2017; Stagg 2018; Balraadjsing *et al*. 2019). This phenomenon is referred to as the silent survival of *S. aureus*, and several studies have provided evidence for a silent migration of *S. aureus* inside immune cells, leading to its dissemination to various parts of the human body (Thwaites and Gant 2011; Rodríguez 2014; Krezalek *et al*. 2018; Zhu *et al*. 2020). Recent studies also hypothesized that MRSA may travel from the gut to a wound via blood cells, for instance neutrophils, thereby causing postoperative wound infection (Krezalek *et al*. 2018; Zhu *et al*. 2020) (Fig. 3).

In recent years, several studies investigated the possible impact of the human gut microbiota on distant organs, including the lungs. Accordingly, the possible cross-talk between the gut microbiota and the lungs seems to have a role in the onset of some lung infections, such as *S. aureus* pneumonia. In fact, the gut–lung axis involves the circulation of lymphocytes, inflammation mediators (e.g. endotoxins), microbial metabolites, cytokines and hormones via the lymph and blood flow, reaching both the lungs and the gut (Fig. 1) (Budden *et al*. 2017; Zhang *et al*. 2020; Sencio, Machado and Trottein 2021). Additionally, the intestinal microbiota is known to balance between pro-inflammatory and regulatory responses, thereby shaping the host's immune system (Belkaid and Hand 2014). This gut–lung interaction can proceed in two ways. First, the gut microbiota may play a direct role in *S. aureus* pneumonia and, second, the presence of *S. aureus* in the gut microbiota may indirectly influence the course of certain respiratory conditions caused by (other) bacteria and viruses (Wang *et al*. 2013; Gauguet *et al*. 2015; Zhang *et al*. 2020; Sencio, Machado and Trottein 2021). The latter idea is supported by the observation that *S. aureus* colonization of the upper respiratory mucosa can decrease influenza-mediated lung immune injury. In fact, *S. aureus* recruits peripheral monocytes into the alveoli, leading to their polarization into M2 alveolar macrophages through Toll-like receptor 2 signaling, which, in turn, will inhibit influenza-mediated inflammation (Wang *et al*. 2013). These observations call for further investigations on the relationships between *S. aureus* gut colonization and respiratory disease development to increase our understanding of the roles of *S. aureus* in the gut–lung axis.

## THE SWITCH BETWEEN COLONIZATION AND DISEASE: THE ROLE OF THE BACTERIA AND THE HUMAN HOST

As described in the previous paragraphs, *S. aureus* plays an important role as a colonizer of the human host since an early age. However, this bacterium is also known for its pathogenicity as a causative agent of mild to more serious skin, soft tissue or surgical site infections, which may also lead to invasive diseases, such as bloodstream infections, endocarditis and sepsis (Wertheim *et al*. 2005; Corey 2009; Anderson and Kaye 2009; Fukuda *et al*. 2020). The relationships between colonization and disease in the human body, and the switch in between the two conditions is a multifactorial and complex process, which is still not fully understood (Mulcahy and McLoughlin 2016). In recent years, several studies suggested that colonization strains can be a potential reservoir for infection, and this hypothesis was based on the observation that strains causing bloodstream infection in patients were clonally identical to the *S. aureus* isolates from the anterior nares of the respective patients (von Eiff and Peters 2001; Sakr *et al*. 2018; Bode *et al*. 2010). Furthermore, it was reported that nasal carriers have a significantly greater risk of contracting bacteremia, and that the majority (>80%) of nosocomial *S. aureus* bacteremia cases are caused by invasion of the endogenous colonizing strain (Brown *et al*. 2014). A study where asymptomatically colonizing *S. aureus* USA300 was tracked at different body sites (nose, throat, perirectal region) after an initial infection showed that clonal isolates of this lineage continued to colonize people up to a year after the initial infection. However, the remaining bacteria experienced the loss or gain of plasmids and mobile genetic elements (e.g. SCC*mec*), or particular mutations in the accessory gene regulator (*agr*) operon (Read *et al*. 2018).

The molecular factors that influence the switch of *S. aureus* from colonizer to pathogen are dependent on both the bacteria and the human body (Table 1) (Brown *et al*. 2014; Mulcahy and McLoughlin 2016; Balasubramanian *et al*. 2017). In general, *S. aureus* is equipped with an adequate repertoire of immune evasive molecules (de Jong, van Kessel and van Strijp 2019; Cheung, Bae and Otto 2021). In addition, under selective pressure the bacteria will acquire genomic variation and display phenotypic changes. The acquired genomic variations may lead to altered virulence, antibiotic resistance and better replication or adaptation to a new anatomical niche of the human host and can be brought about by SNPs or mobile genetic elements, such as bacteriophages, plasmids and transposons (Young *et al*. 2012; Fitzgerald 2014; Lindsay 2014; Messina *et al*. 2016; Giulieri *et al*. 2018; Guérillot *et al*. 2019). Different *S. aureus* strains display distinct expression of virulence factors, which are the key players for survival at different body sites and pathogenesis (Zhao *et al*. 2019, 2020). These virulence factors can be either bacterial cell surface-associated or secreted proteins (Dreisbach *et al*. 2020). Virulence factors have disparate roles, which can promote immune evasion, adhesion and invasion of the host cells, or host cell injury and cell death (Sibbald *et al*. 2006; de Jong, van Kessel and van Strijp 2019). For example, several *S. aureus* toxins are involved in disease pathogenesis, including pore-forming toxins (PFTs), α-toxin and the bicomponent leukocidins, which bind to membrane-associated receptors in the host cells. These toxins show differences in host cell lysis, which can be attributed to cell type specificity in toxin binding and synergies between different toxins (Berube and Bubeck Wardenburg 2013; Seilie and Bubeck Wardenburg 2017; Spaan, van Strijp and Torres 2017). The acquisition of genomic mutations can also be related to a more invasive behavior. For example, SNPs in the fibronectin-binding protein A (FNBPA), which binds to human fibronectin, were linked to an increased risk of cardiac device infection (Hos *et al*. 2015). Another study showed that some strains that colonize and infect the human skin present mutations (SNPs) in metabolic genes like the *fumC* gene for class II fumarate hydratase (Acker *et al*. 2019). The transition of an asymptomatically carried MSSA population to a fatal bloodstream infection was shown to be associated with only few mutations, found for example in the AraC transcriptional regulator of stress response and pathogenesis (Young *et al*. 2012). A possible causal relationship between genetic mutations with biofilm formation and with infection was shown in recent works based on the rise of mutations in the *agr* genes (Suligoy *et al*. 2018; Tan *et al*. 2018; Gor *et al*. 2019). For example, it was shown that some Agr-negative strains are phase variants due to reversible genetic mutations in the *agr* locus and that these Agr-negative strains are able to revert their Agr phenotype (Gor *et al*. 2019). These findings can be related to the hypothesis that over a period of time, *S. aureus* colonizing the human host will acquire genetic variations associated with infection at the colonization site. This will then lead to the emergence of bacteria causing infection phenotypes when an unknown trigger is perceived by the bacteria (Fitzgerald 2014). For this reason, when an infection occurs, it is interesting to know what type of host immune failures occurred to allow *S. aureus* invasion and to identify the nature of the unknown trigger.

**Table 1.** Determinants for the *S. aureus* switch from colonizer to pathogen.

Bacterial factors	Human factors	Coexisting microbiota	Other triggers	Time
– **Virulence factors**; e.g. regulators of gene expression, surface-associated and secreted virulence factors, and small molecules – **MGEs**; e.g. bacteriophages, pathogenicity islands, staphylococcal cassette chromosomes; plasmids and transposons – **Variations in the bacterial genome**; e.g. clonal variations, gene level variations and single nucleotide variations – **Metabolic adaptations to different niches of the human body**; e.g. for adaptation to the nasal environment and intracellular adaptation – **Bacterial load**; e.g. by influencing the immune clearance, defining a persistent or intermittent carrier	– **General factors**; e.g. sex, gender, age, ethnicity, previous or current diseases – **Innate and adaptive immune systems** including immune history – **The immune imprint by the bacteria and the host response during asymptomatic periods of colonization**; e.g. immunoregulation of *S. aureus* during nasal colonization – **Variations in the human genome**; e.g. SNPs encoding factors involved in local immunity or congenital loss-of-(complete)-function mutations that lead to neutrophil-related disorders	– **Coresident microbiota**; e.g. other bacteria present in the nasopharynx, lungs, the gut or (chronic) wounds – **Changes in the microbiota that modulate *S. aureus* virulence or colonization**	– **Physical triggers**; e.g. trauma, burns or surgical wounds – **Factors that affect integrity of the human body barriers and homeostasis**; e.g. dysbiosis – **Contacts with livestock**; livestock-associated MSSA or MRSA – **Unknown triggers;** e.g. nutritional status and lifestyle	– **The development of infection can occur after different periods of time**; e.g. time after surgery

With respect to human factors involved in the switch from colonizer to pathogen, it is important to consider the host variations in the response to infection due to the state of the immune system, previous diseases, the immune history, sex, interactions with other pathogens in the human body and SNPs in specific human genes (Table 1) (Ruimy *et al*. 2010; Sollid *et al*. 2014; Messina *et al*. 2016; Mulcahy and McLoughlin 2016). For example, human leukocyte antigen (HLA) class II polymorphisms determine the response to bacterial superantigens, which is also related to a different T-cell proliferation and cytokine production (Shepherd and McLaren 2020). An epidemiological and microbiological study highlighted that the predominant factor determining persistent colonization by *S. aureus* was apparently a specific set of genetic polymorphisms in the host genes for the C-reactive protein (CRP) and interleukin 4 (IL-4) (Shepherd and McLaren 2020). Additionally, the presence of SNPs in cytokine genes, such as IL6, TNF, IL10, IL17A, IFNG and in the inhibitory toll-like receptor TLR10, seem to play a role in the susceptibility to complicated skin infections (Stappers *et al*. 2014, 2015). Important differences between nasal *S. aureus* carriers and noncarriers were related to polymorphisms in soluble or membrane-bound molecules, such as TLR9, the glucocorticoid receptor and the β-defensin 1 (Sakr *et al*. 2018). Several pathological conditions affecting the immune system, such as leukopenia, were shown to lead to a different *S. aureus* disease severity and infection outcome (Khanafer *et al*. 2013). Lastly, a study in mice unveiled the important role of neutrophil influx in the depletion of *S. aureus* from sites of infection (Archer, Harro and Shirtliff 2013).

Recent studies have shown that both the immune imprint of the bacteria and the host-responses during an asymptomatic period of colonization, involving both the activation of the innate immune responses and of cell-mediated adaptive immune responses, seem to be very important for the human host (Verkaik *et al*. 2009; Brown *et al*. 2014; Teymournejad and Montgomery 2021). In addition, the interplay with the coexisting microbiota also influences colonization and immune regulation. In a study on patients with *S. aureus* bacteremia, distinctive patterns in the human antibody response to endogenous versus exogenous infection were observed between carriers and noncarriers (Kolata *et al*. 2011). Several studies showed heterogeneity in the humoral immune response against different staphylococcal antigens among *S. aureus* carriers and noncarriers (Dryla *et al*. 2005; Verkaik *et al*. 2009; Ghasemzadeh-Moghaddam *et al*. 2017). In nasal carriers, lower mortality rates were observed upon *S. aureus* bacteremia compared with noncarriers. This could be linked to a crosstalk of the bacteria and the immune system during colonization, resulting in an immunological advantage (Mulcahy and McLoughlin 2016). A low Th1 to Th17 cytokine mRNA ratio was shown to be predictive of *S. aureus* carriage in volunteers after whole blood stimulation (Nurjadi *et al*. 2016). Neonatal mucosal colonization by *S. aureus* strains with certain combinations of genes specifying superantigens and adhesins may result in immune stimulation, which, in turn, can result in a strengthening of the epithelial barrier that counteracts the development of atopic eczema (Nowrouzian *et al*. 2019). Together, these studies imply a close connection between the host responses during colonization and the subsequent development of infection in response to a ‘trigger’. Future human studies should therefore be conducted to investigate the role of the immune imprint of *S. aureus* during gut colonization next to the nasal colonization and the subsequent development of infection. This could help answering the question of whether colonization with *S. aureus* may actually have certain advantages for the human host, particularly by modulating the course of *S. aureus* infection, or even infection by other bacterial or viral pathogens.

*S. aureus* is a known causative agent of postoperative wound infections and infections of implants in the human body (Fig. 3). In fact, MRSA is one of the leading bacteria causing surgical site infections (Fukuda *et al*. 2020; Anderson and Kaye 2009). *Staphylococcus aureus* preponderates in orthopedic or cardiac surgery settings, where biofilm can form on implanted materials at different time intervals after the surgery. Early onset infections may be a consequence of contamination during surgery. However, some studies have shown that in certain patients who develop MRSA infections, wound cultures did not reveal intraoperative MRSA contamination at the time of wound closure immediately after the surgery (Morton *et al*. 2016; Krezalek *et al*. 2018; Zhu *et al*. 2020). This is suggestive of infections through another route. In fact, endogenous carriage of MRSA was shown to be a risk factor for the development of surgical site infections, but how the pathogen travels to the surgical site is still controversial. Several studies have proposed a relationship with blood cells, such as neutrophils, that could serve as vectors to carry *S. aureus* to the site of the surgical wound (Thwaites and Gant 2011; Greenlee-Wacker *et al*. 2014; Krezalek *et al*. 2018; Zhu *et al*. 2020). This hypothesis could be extended also to other immune cells, as it is known that *S. aureus* is not only internalized by the relatively short-lived neutrophils, but also by cells with a longer lifetime like monocytes, macrophages and dendritic cells (Kubica *et al*. 2008; Horn *et al*. 2017; Balraadjsing *et al*. 2019). The mobile immune cells can move within localized or extended areas of the human body, thereby leading to dissemination of the *S. aureus* infection (Kubica *et al*. 2008; Thwaites and Gant 2011). Future studies, including the labeling of infected immune cells and tracking their possible migration to surgical sites in an appropriate animal model could be conducted to obtain a better understanding of the possible spread of infection through the movement of immune cells with intracellular bacteria to sites of inflammation (Krezalek *et al*. 2018; Zhu *et al*. 2020). In recent years, various tools have been developed, which may allow to perform such investigations. These include fluorescently labeled antibiotics (e.g. vancomycin), or monoclonal antibodies that specifically target *S. aureus* (van Oosten *et al*. 2013; Romero Pastrana *et al*. 2018; Zoller *et al*. 2019; Park *et al*. 2021). However, the limitation of these probes is that they mainly recognize extracellular bacteria, so they would allow the detection of bacteria only once they are released from the silent carrier at new colonization sites or sites of infection. Nanoparticle-based probes were reported to allow improved intracellular detection and they may display enhanced bactericidal activity (Hussain *et al*. 2018; Zhou *et al*. 2018). Another parameter that needs to be taken into consideration is the signal emitted by the probe and the imaging tool that allows its visualization. For bacterial detection in tissues or infected cells *ex vivo* several fluorescence-based approaches, such as microscopy or flow cytometry, have been used in different studies (Krezalek *et al*. 2018; Zhu *et al*. 2020). *In vivo* experiments to track the bacterial migration inside human cells are more challenging as it requires a technique that allows to image the bacteria through different tissues. In particular, fluorescent light has a tissue penetration of up to ∼10 mm, depending on the wavelength (van Oosten *et al*. 2015; Ordonez *et al*. 2019). Nuclear imaging techniques, such as positron emission tomography (PET), take advantage of the fact that the emitted radiation by PET tracers has a very high tissue penetration (Ordonez and Jain 2018). Another parameter that needs to be taken into consideration is the bacterial load that needs to be detected as this will influence the signal intensity and the distribution of the emitted signal across the body of an experimental animal. Several options are currently explored for the noninvasive detection of staphylococcal infections, and preclinical studies have shown that this is highly feasible in the case of infections of the skin, muscles and implanted biomaterials. However, further advances with respect to sensitivity and resolution are needed, before these techniques can also be employed to visualize silent intracellular trafficking of *S. aureus* inside blood cells. Perhaps the currently most feasible approach would be to collect immune cells from an experimental animal, infect these cells *in vitro* with bacteria that have been labeled with a PET tracer, reintroduce the infected immune cells into the animal at different body sites and follow the fate of the bacteria using a sensitive micro-PET system.

In the context of surgical site infections and infections of implanted medical devices, it is also important to consider the interaction between *S. aureus* and the related bacterium *Staphylococcus epidermidis. Staphylococcus epidermidis* is part of the human microbiota, colonizing mostly the mucosa and skin. This bacterium is particularly well adapted for colonization of the relatively dry niches of the human skin, because it can withstand conditions with low water activity (de Goffau *et al*. 2009; Goffau, van Dijl and Harmsen 2011). However, it was shown that skin colonization by *S. epidermidis* is not entirely symbiotic for the human host, since particular strains of *S. epidermidis* can cause infection and may even modulate *S. aureus* colonization (Sabaté Brescó *et al*. 2017; Brown and Horswill 2020; Du *et al*. 2021). For instance, *S. epidermidis* is infamous for its tight adherence to implanted medical devices and the formation of thick biofilms that are hard to eradicate by antimicrobial therapy. Moreover, the dispersal of *S. epidermidis* bacteria from biofilms on medical implants was shown to cause bacteremia. The high ability of *S. epidermidis* to easily form biofilms is a major reason why this bacterium is a predominant cause of postoperative infections (Nguyen, Park and Otto 2017; Sabaté Brescó *et al*. 2017). In contrast to *S. aureus*, which produces a broad range of different virulence factors, *S. epidermidis* has a relatively limited repertoire of virulence factors and, accordingly, it displays a much lower invasive behavior (Namvar *et al*. 2014; Nguyen, Park and Otto 2017; Sabaté Brescó *et al*. 2017). For example, compared with *S. aureus* the toxin production by *S. epidermidis* is mostly limited to phenol-soluble modulins (PSMs). In fact, the most important facilitators of *S. epidermidis* pathogenicity are molecules promoting adhesion to native and protein-coated surfaces, and factors necessary for the formation and maturation of biofilms (Otto 2009; Namvar *et al*. 2014; Büttner, Mack and Rohde 2015; Sabaté Brescó *et al*. 2017; Du *et al*. 2021). Additionally, *S. epidermidis* was shown to form small colony variants (SCVs) with lowered metabolic activity upon internalization by host cells, allowing intracellular survival and persistence (Kahl, Becker and Löffler 2016; Sabaté Brescó *et al*. 2017). However, the mechanisms of intracellular *S. epidermidis* persistence are less well characterized than those of *S. aureus*. Still, intracellular persistence of *S. epidermidis* was demonstrated for dendritic cells, macrophages, fibroblasts and osteoblasts (Sabaté Brescó *et al*. 2017; Magryś *et al*. 2018; Balraadjsing *et al*. 2019; Fisher and Patel 2020). The latter studies also showed that intracellular *S. epidermidis* can reside in phagolysosomes and escape into the extracellular environment upon host cell death (Magryś *et al*. 2018; Perez and Patel 2018). It was also proposed that this mechanism could lead to the formation of biofilms on implants and cause late-onset implant-associated infections (Perez and Patel 2018).

## *STAPHYLOCOCCUS AUREUS* AND INNATE IMMUNE CELLS: THE STRATEGIES FOR SURVIVAL AND BACTERIAL DISSEMINATION

The interaction between *S. aureus* and immune cells in different parts of the human body is fundamental not only during infection of tissues and the bloodstream, but also during colonization of the nasopharynx, gut and lungs. These interactions may in fact enhance *S. aureus* virulence, internalization or colonization, or they may promote other cellular activities and inflammation (Table 2). During its evolution, *S. aureus* evolved multiple factors to help evade the innate immune defenses and to colonize the human host. In the first line of defense, innate immune cells play fundamental roles in detecting and mediating bacterial infection. In fact, cells such as granulocytes (basophils, neutrophils and eosinophils), dendritic cells, monocytes, macrophages, neutrophils and natural killer cells have a fundamental role in disease development (Greenlee-Wacker *et al*. 2014; Flannagan, Heit and Heinrichs 2015; Melehani *et al*. 2015; Berends *et al*. 2019). Additionally, blood cells with their movement throughout the human body can facilitate *S. aureus* intracellular survival, leading to dissemination to other body sites and development of innate immune memory (Kubica *et al*. 2008; Thwaites and Gant 2011; Mulcahy and McLoughlin 2016; Krezalek *et al*. 2018; Zhu *et al*. 2020). Even platelets can play roles that impact on *S. aureus* survival (Ali *et al*. 2017).

**Table 2.** Overview of possible interactions between *S. aureus* and innate immune cells.

Immune cells	Interaction with *S. aureus*	Immune cell-mediated *S. aureus* killing	*S. aureus* immune evasion	*S. aureus* intracellular trafficking and infection development
**Neutrophils**	– **Life span**: ≅few hours to several days – **Body sites**: vasculature, oral and nasal cavity, gut and lungs – **Movement**: highly mobile	– **Extracellular antimicrobial killing**: neutrophil extracellular traps (NETs) and degranulation – **Phagocytosis and bacterial killing**	– *S. aureus* from the extracellular environment evades the immune cells’ killing mechanisms and can mediate cell lysis – *S. aureus* survival within the phagosome and/or intracellular replication – *S. aureus* escapes from the phagosome, proliferates in the cytosol, causes host cell lysis and escapes	– Thwaites and Gant (2011) – Krezalek *et al*. (2018) – Zhu *et al*. (2020)
**Monocytes**	– **Life span**: relatively brief, until ≅24 h – **Body sites and movement**: mobile in the vasculature	– **Extracellular antimicrobial killing** – **Phagocytosis and bacterial killing**	– *S. aureus* from the extracellular environment evades the immune cells’ killing mechanisms and can mediate cell lysis – *S. aureus* intracellular survival	– No published evidence for *S. aureus* – Publications on other pathogens (e.g. *Listeria, Mycobacterium*)
**Macrophages**	– **Life span**: relatively long, from months to years – **Body sites**: oral and nasal cavity, gut and lungs – **Movement**: limited within the tissues	– **Extracellular antimicrobial killing**: METs and degranulation – **Phagocytosis and bacterial killing**	– *S. aureus* from the extracellular environment evades the immune cells’ killing mechanisms and can mediate cell lysis – *S. aureus* survival within the phagosome and/or intracellular replication – *S. aureus* escapes from the phagosome, proliferates in the cytosol, causes host cell lysis and escapes	– No published evidence for *S. aureus*
**Dendritic cells**	– **Life span**: ≅10 days – **Body sites and movement**: mobile in the vasculature	– **Bacterial uptake**: cause bacterial lysis and present bacteria-derived peptides on MHC class II molecules to T cells and initiate specific immune responses	– *S. aureus* from the extracellular environment evades the immune cells’ killing mechanisms and can mediate cell lysis – *S. aureus* escapes from the phagosome, persists intracellularly or is released into the cytosol and subsequently into the extracellular environment	– No published evidence for *S. aureus* – Evidences for other pathogens (e.g. *Salmonella, Mycobacterium*)
**Platelets**	– **Life span**: until ≅15 days – **Body sites and movement**: highly mobile vasculature, oral and nasal cavity, gut and lungs	– **Direct antimicrobial activity** by killing extracellular bacteria, or **indirect antimicrobial activity** by modulating immune responses in different ways	– *S. aureus* from the extracellular environment can inhibit and modulate platelet function	– No evidence and unlikely due to size constraints
**Natural killer cells**	– **Life span**: until ≅15 days – **Body sites and movement**: highly mobile vasculature, oral and nasal cavity, gut and lungs	– **Direct antimicrobial activity** by killing the bacteria or the infected cells – **Indirect antimicrobial activity** by stimulating the activity of other immune cells	– *S. aureus* from the extracellular environment evades the immune cells’ killing mechanisms, can manipulate the host cells and can cause host cell lysis	– No published evidence for *S. aureus* or other bacterial pathogens

Neutrophils are the primary mediators of the innate host defenses against bacterial, viral and fungal pathogens that take place before the more complex humoral and lymphocyte cellular processes of acquired immunity can act against an infection. The key functions of neutrophils are chemotaxis, phagocytosis, production of reactive oxygen species (ROS), production of cytokines/chemokines, secretion of peptides and enzymes during the process of degranulation, and release of neutrophil extracellular traps (NETs) (Spaan *et al*. 2013; Malech, DeLeo and Quinn 2014). The neutrophils are produced both from progenitors in the bone marrow and certain extramedullary tissues. When neutrophils mature, they exist primarily as free-flowing in the intravascular blood pool. However, after activation, neutrophils migrate from the vasculature through the blood vessel to a site of infection (Rigby and DeLeo 2012). In fact, neutrophils continuously transmigrate trough the junctional epithelium protecting the oral mucosal barrier, but they can also be rapidly recruited to the nasal airways in case of infection (Moutsopoulos and Konkel 2018; Ge *et al*. 2020). Additionally, neutrophils are the sentinels that can kill luminal gut bacteria if they translocate across the epithelium and invade the mucosa. Neutrophils can also migrate to the apical surface of the lung epithelium and, upon transepithelial migration, they can eliminate invading pathogens (Fournier and Parkos 2012; Adams, Espicha and Estipona 2021). Lastly, neutrophil influx is also essential for bacterial clearance during cutaneous wound healing (Kim *et al*. 2008). *Staphylococcus aureus* can interact with the neutrophils at different body sites, such as the bloodstream, or the tissues of the skin, nose, mouth, gut and lungs (Kim *et al*. 2008; Thwaites and Gant 2011; Uriarte, Edmisson and Jimenez-Flores 2016; Ge *et al*. 2020; Zhu *et al*. 2020). When *S. aureus* is opsonized either by complement and/or immunoglobulins, this may lead to phagocytosis of the bacteria. However, *S. aureus* can effectively evade the different immune defense mechanisms, such as neutrophil recruitment, chemotaxis, priming, activation, production of ROS and neutrophil effector functions, cell lysis and apoptosis (Guerra *et al*. 2017; Kobayashi, Malachowa and DeLeo 2018; Cheung, Bae and Otto 2021). For example, this bacterium can prevent the neutrophils from migrating to the site of infection through the secretion of superantigen-like proteins 5 and 10 (SSL5 and SSL10), formyl peptide receptor-like inhibitory proteins (FLIPr and FLIPr-like) and the chemotaxis inhibitory protein of *S. aureus* (CHIPS) (Cole *et al*. 2001). Additionally, the evasion of neutrophil killing is achieved by regulated expression of virulence factors, bacterial cell membrane modifications or the production of particular enzymes. For example, *S. aureus* targets bactericidal mechanisms that follow phagocytosis with proteases such as aureolysin, with proteins such as staphylokinase, with superoxide dismutases such as SodA and SodM, and with catalases such as KatA (Guerra *et al*. 2017). Additionally, *S. aureus* is able to cleave neutrophil-derived antimicrobial peptides rendering them inactive, to produce nucleases that degrade the NETs and allow escape from these DNA traps, and also to trigger the caspase-3-mediated death of neutrophils (Wertheim *et al*. 2005). Production of toxins, including leukocidins that bind to specific receptors on the immune cells is also a mechanism of immune evasion, which may lead to neutrophil lysis (Spaan, van Strijp and Torres 2017). The effects of Panton-Valentine leukocidin (PVL), LukED, HlgAB, HlgCB and LukAB have been studied for different types of immune cells (DuMont *et al*. 2013; Spaan, Henry *et al*. 2013; Melehani *et al*. 2015; Spaan, van Strijp and Torres 2017; Tromp *et al*. 2018). In contrast to PVL, PSMs are also important toxins of *S. aureus* and they are not species specific. There are four types of PSMs known in *S. aureus*, namely the PSMα, PSMβ, PSMmec and PSMγ. It was shown that the PSMα proteins have cytolytic activity towards neutrophils, particularly PSMα3 (Wang *et al*. 2007; Surewaard *et al*. 2013). Another important feature of PSMα is that, if there is enough intracellular production of PSMα in the phagosome, it will cause neutrophil lysis and bacterial survival, which will contribute to bacterial dissemination (Grosz *et al*. 2014). Consistent with these staphylococcal immune evasion mechanisms, it was observed that individuals with congenital defective mutations that lead to severe neutropenia, neutrophil granule disorders, defective neutrophil chemotaxis or defective ROS-mediated killing, were apparently more sensitive to *S. aureus* infections (Bouma *et al*. 2010; Miller and Cho 2011; Miller *et al*. 2020). As neutrophils are mobile elements that can rapidly transmigrate from the bloodstream to deeper tissues, in some studies it was proposed that they may even represent a protective niche where *S. aureus* could hide to evade antimicrobial therapy, and that they may also serve as a trojan horse by which the bacterium can travel from the bloodstream to surgical sites causing an infection (Thwaites and Gant 2011; Krezalek *et al*. 2018; Zhu *et al*. 2020). Lastly, it is also important to consider the life span of neutrophils to better understand their possible role as trojan horses. In fact, several studies focused on neutrophil kinetics in peripheral blood, using radioactive or stable isotope labeling, which showed that their life span can vary from a few hours to several days (Hidalgo *et al*. 2019). However, even though the mechanisms of survival and the presence of intracellular *S. aureus* inside neutrophils were clearly demonstrated *in vitro*, for example in studies using polymorphonuclear neutrophils, and *ex vivo* using tissues from patients or from animal models, only few studies focused on the trafficking and the role of neutrophils as an intracellular reservoir that leads to the development of infections *in vivo* (Gresham *et al*. 2000; Thwaites and Gant 2011; Greenlee-Wacker *et al*. 2014; Horn *et al*. 2017; Moldovan and Fraunholz 2019). Recent, studies involving animal models, showed the silent trafficking of intracellular MRSA in neutrophils from the gut environment to the wound, without the development of sepsis or bacteremia, thereby causing postoperative wound infection or prosthetic joint infection (Krezalek *et al*. 2018; Zhu *et al*. 2020).

Monocytes are bone marrow-derived leukocytes, which move into the bloodstream and can migrate into tissues and differentiate into monocyte-derived macrophages or monocyte-derived dendritic cells. These cells have the ability to balance between tolerance and immunity. In fact, once an infection occurs monocytes are recruited into the bloodstream and they play a role both in the inflammatory and anti-inflammatory processes that take place during the immune response (Serbina *et al*. 2008; Guilliams *et al*. 2014; Xiong and Pamer 2015). The extravasation of monocytes from the bloodstream leads to an immune cell population composed of monocytes, tissue-resident macrophages and intestinal or lung dendritic cells. The intestinal dendritic cells are concentrated in the lamina propria of the gut while, in the human respiratory tract, alveolar or interstitial macrophages and lung dendritic cells are encountered (Coombes and Powrie 2008; Bain and Mowat 2014; Kopf, Schneider and Nobs 2015). Additionally, the oral and nasal mucosal barriers harbor dendritic cells, macrophages and recruited monocytes that have specific roles in protecting the mucosa against bacterial infections (Zhang *et al*. 2016; Moutsopoulos and Konkel 2018). *Staphylococcus aureus* can interact with monocytes in the bloodstream, but also with macrophages and dendritic cells in the tissues of the skin, the nasal and oral cavities, the gut and the lungs (Balraadjsing *et al*. 2019; Musilova *et al*. 2019; Kearney *et al*. 2020; Pidwill *et al*. 2021). Altogether, monocytes and macrophages are involved in phagocytosis and intracellular killing of microorganisms. When *S. aureus* is confronted by these cells, the bacteria may be killed by several mechanisms, either extracellularly through capture in macrophage extracellular traps (mETs), degranulation and the action of antimicrobial peptides and ROS, or intracellularly in phagosomes through the concerted actions of ROS, reactive nitrogen species (RNS), acidic pH, enzymes and nutrient restriction (i.e. ‘nutritional immunity’) (Flannagan, Heit and Heinrichs 2015; Pidwill *et al*. 2021). On the contrary, extracellular *S. aureus* can kill macrophages or employ different escape mechanisms to survive phagocytosis. For example, *S. aureus* can survive in subcellular organelles of macrophages, especially phagosomes and vacuoles, without affecting the viability of the cells, but the bacterium may also replicate intracellularly and cause death of the macrophage (Kubica *et al*. 2008; Pidwill *et al*. 2021). The *S. aureus* bacteria that have escaped from the macrophages can travel through the bloodstream and may cause infection at other body sites. Additionally, the presence of *S. aureus* may influence macrophage polarization and secretion of either pro-inflammatory cytokines or anti-inflammatory cytokines (Flannagan, Heit and Heinrichs 2015; Flannagan, Heit and Heinrichs 2016; Chan *et al*. 2018; Feuerstein *et al*. 2020; Flannagan and Heinrichs 2020; Pidwill *et al*. 2021). In particular, the monocyte immune response in terms of pro-inflammatory cytokine production was shown to be lowered by MRSA with the sequence type ST80 (Kolonitsiou *et al*. 2019). The immune evasion mechanisms that *S. aureus* can employ to evade macrophage function are very diverse, ranging from host cell intoxication with leukotoxins (e.g. PSMs, leukocidins and hemolysins), avoidance of phagocytosis by complement inhibition or opsonin interference, bacterial cell surface modifications, high resistance to ROS and RNS through the production of antioxidant activities (e.g. staphyloxanthin and the lactate dehydrogenase Ldh1), to the overcoming of nutritional immunity by the capture of nutrients from the host (Koziel *et al*. 2009; Loffler *et al*. 2010; Thomsen *et al*. 2014; Flannagan, Heit and Heinrichs 2015). Lastly, it was observed that *S. aureus* can activate a TLR2-dependent endosomal signaling pathway upon internalization by monocytes, which allows the bacterium to use the host signaling for its own proliferation in the human body (Musilova *et al*. 2019). The survival of *S. aureus* over time inside monocytes and macrophages may allow the bacteria to withstand antibiotic therapy, which can subsequently lead to a relapse of infection and bacterial dissemination (Kubica *et al*. 2008; Thwaites and Gant 2011; Lacoma *et al*. 2017; Peyrusson *et al*. 2020). In fact, intracellular *S. aureus* persister cells in monocytes and macrophages were shown to remain metabolically active, and to display an altered transcriptomic profile associated with multidrug tolerance upon antibiotic exposure (Peyrusson *et al*. 2020). As pointed out above for neutrophils, the life span of blood cells is an important parameter to consider when studying *S. aureus* intracellular survival and dissemination. In this respect, it is noteworthy that the life of monocytes is very short (∼24 h), while macrophages have a longer life span, ranging from months to years (Guerra *et al*. 2017; Patel, Ginhoux and Yona 2021). Interestingly, there is so far no published evidence that monocytes and macrophages could be involved in the silent intracellular trafficking of *S. aureus*, as was shown for neutrophils.

Dendritic cells are bone marrow-derived leukocytes, which circulate in the bloodstream and subsequently reach lymphoid organs (e.g. the spleen, thymus and lymph nodes) as well as nonlymphoid organs (e.g. the skin). In the skin, dendritic cells can mature and then enter the lymphatic vasculature to be transported to the lymph nodes, where they may present antigens to B and T cells. Migration is a key important feature of the dendritic cells. Additionally, dendritic cells form a family of antigen-presenting cells that are present in almost all tissues of the body, where they serve to capture bacteria and other pathogens. Subsequently, these dendritic cells present the antigens of the captured pathogens to initiate tolerogenic immune responses. For this reason, dendritic cells are described as ‘immune saviors’ that form a connection between the innate and adaptive immune systems, inducing both primary and secondary immune responses (Geissmann 2007; Liu and Nussenzweig 2010; Worbs, Hammerschmidt and Förster 2017). Intestinal dendritic cells are responsible for establishing tolerance towards the microbiota, but also initiating immune responses against mucosal pathogens (Sun, Nguyen and Gommerman 2020). Also in the mucosa of the lungs, oral cavity and nasal cavity, the dendritic cells play important roles in the protection against pathogens and the development of tolerogenic immune responses (Cutler and Jotwani 2006; Lee *et al*. 2015; Cook and MacDonald 2016). Compared with human monocytes and macrophages, human dendritic cells kill internalized pathogens at relatively low efficiency. Nonetheless, dendritic cells are able to take up *S. aureus*, lyse the bacteria and present bacteria-derived peptides on major histocompatibility complex (MHC) class II molecules to T cells and initiate a specific immune response (Darisipudi *et al*. 2018; Balraadjsing *et al*. 2019). However, it was shown that *S. aureus* can mount diverse defensive mechanisms to avoid opsonization, phagocytosis and proteolytic degradation by dendritic cells, and that *S. aureus* manipulates the dendritic cells with the final aim of surviving their insults (Darisipudi *et al*. 2018). For example, *S. aureus* can evade or modulate dendritic cell responses by intensifying their pro-inflammatory response in an antigen nonspecific manner through the production of superantigens (SAgs) that cross-link T cell receptors with MHC class II molecules on the dendritic cells. In turn, this may lead to higher pro-inflammatory cytokine production and a status of shock or cell death (Voorhees *et al*. 2011; Schindler *et al*. 2012; Balraadjsing *et al*. 2019). Furthermore, *S. aureus* produces several pore-forming toxins, such as leukocidins, that can directly kill dendritic cells or diminish dendritic cell-mediated activation of CD4^+^ T lymphocytes, thereby weakening the development of adaptive immunity (Darisipudi *et al*. 2018; Berends *et al*. 2019). Intracellularly, *S. aureus* can escape from the phagosomes of dendritic cells, to be released into the cytoplasm and subsequently the extracellular environment. However, *S. aureus* can also change the pH of the phagosomes by producing urease and preventing their lysis (Bore *et al*. 2007; Darisipudi *et al*. 2018). Therefore, the possibility of silent intracellular presence of *S. aureus* in dendritic cells may be considered as a means of survival and dissemination, especially since these cells are highly mobile inside the human body, circulating in the blood and lymphatic system. Although not yet demonstrated for *S. aureus*, some studies have evidenced the physiological translocation of nonpathogenic bacteria from the gut lumen, via dendritic cells and CD18^+^ cells, to other locations in the body, including lactating mammary glands (Rodríguez 2014). Additionally, dendritic cells were shown to represent a niche for other bacterial pathogens during the early stages of infection and for the subsequent pathogen dissemination (Bar-Haim *et al*. 2008; Reizis 2011; Aulicino *et al*. 2018). Lastly, various labeling approaches have shown that the rates of survival of different dendritic cell subsets from different lymphoid organs can vary substantially, but with a maximum survival of 14 days (Kamath *et al*. 2002). In view of this relatively long survival period, combined with the possibility of intracellular survival of *S. aureus*, it seems important to consider also dendritic cells as potential trojan horses for this pathogen.

Platelets play an important role in hemostasis and immunity. These cells circulate in blood, surveying the vasculature for hemostatic and immune threats. In fact, platelets interact with the leukocytes and have a role in both the innate and adaptive immune responses. These anucleate cells are relatively short-lived, as they can last only around 10 days before being removed in the liver and spleen. Platelets can modulate the inflammatory response in different ways, especially by expressing TLRs, promoting NETs formation by neutrophils, promoting or decreasing the activity of other immune cells of the innate and adaptive immune systems, by inducing thrombocytopenia, and by secreting cytokines and chemokines (Kapur *et al*. 2015; Ali *et al*. 2017; Li, Zarbock and Hidalgo 2017; Deppermann and Kubes 2018). For example, platelets can express immunoreceptors and they have the capacity to store various types of bioactive and inflammatory molecules that are released upon their activation following endothelial injury. The latter molecules are stored as granules, including the dense (δ-), alpha (α-) or lysosomal (λ-) granules (Smyth *et al*. 2009). Additionally, they have a direct effector function against the invading microbes through complex receptor–ligand interactions. Examples of these receptors are the complement receptors FcγRIIa, TLRs, GPIIb-IIIa and GPIb (Hamzeh-Cognasse *et al*. 2015). *Staphylococcus aureus* interacts with platelets in the vasculature and platelets can protect the host against *S. aureus* infection and bacteremia, for instance by directly killing the bacteria in a thrombin-dependent manner, which appears to be an actin-dependent process (Wuescher, Takashima and Worth 2015; Ali *et al*. 2017). Of note, the bactericidal activity of platelets seems to be independent of reactive oxygen metabolites (Ali *et al*. 2017). Additionally, the platelets may manage to enhance phagocytosis, restrict the intracellular replication of *S. aureus* in macrophages through IL-1β, and round up the bacteria and force them into clusters, thereby promoting easier recognition and engulfment by macrophages (Ali *et al*. 2017). However, some bacterial factors induce the inhibition of platelet function, such as the staphylococcal enterotoxin B, extracellular fibrinogen-binding protein and staphylokinase (Hamzeh-Cognasse *et al*. 2015). The *S. aureus* α-toxin, which binds to the receptor ADAM10, alters platelet activation and induces neutrophil inflammatory pathways that effect severe human sepsis (Powers *et al*. 2015). No evidence for intracellular survival of *S. aureus* inside platelets or platelet-mediated silent bacterial dissemination and release to other body sites was so far reported. However, the role of platelets is fundamental for the interaction between *S. aureus* and other immune cells, such as macrophages, neutrophils and dendritic cells (Johansson, Shannon and Rasmussen 2011; Ali *et al*. 2017; Nishat, Wuescher and Worth 2018).

Natural killer cells belong to the lymphocytes of the innate immune system that control microbial infections by limiting their spread and subsequent tissue damage. These cells have a regulatory role in the interactions with dendritic cells, macrophages, T cells and endothelial cells with the final outcome of limiting or increasing the immune responses. The natural killer cells are produced in the bone marrow, subsequently access the lymphatic circulation and then spread throughout the lymphoid and nonlymphoid tissues. Of note, these cells can also develop and mature in secondary lymphoid tissues, such as the tonsils, spleen and lymph nodes (Vivier *et al*. 2008; Vogel *et al*. 2014; Abel *et al*. 2018). Natural killer cells can also reach the bloodstream and be disseminated to the lungs, the gut, and the nasal and oral cavities via this route. The life span of natural killer cells in the human body is around 15 days (Vogel *et al*. 2014). Natural killer cells are activated directly or indirectly by interactions with other immune cells, cytokines and bacteria. In fact, natural killer cells can either exert a noncytolytic control of pathogen replication, or display a direct microbicidal activity towards different bacteria or infected bacterial cells through different mechanisms, including the secretion of molecules stored in cytotoxic granules, production of antimicrobial peptides and the activation of death-inducing receptors in other cells with internalized bacteria (Zucchini *et al*. 2008; Schmidt *et al*. 2016). *Staphylococcus aureus* can interact with natural killer cells at the different afore-mentioned body sites where they exert a sentinel role (Kamoda *et al*. 2008; Small *et al*. 2008; Reinhardt *et al*. 2015; Johansson *et al*. 2016; Nowicka 2018; Theresine, Patil and Zimmer 2020; Jang *et al*. 2021). As for other leukocytes, *S. aureus* is able to evade and manipulate natural killer cells. For example, pore-forming leukocidins, such as LukED, were shown to target natural killer cells (Reyes-Robles *et al*. 2013). The bicomponent pore-forming toxins (HlgAB and HlgCB) encoded by *hlg* genes were also shown to have activity towards natural killer cells and, in fact, an HlgABC challenge caused the lysis of natural killer cells (Hodille *et al*. 2020). Also, *S. aureus* β-hemolysin directly upregulates the expression of IFN-γ in human natural killer cells, and this may actually contribute to the pathogenesis of *S. aureus* (Guan *et al*. 2021). However, no evidence of natural killer cell-mediated silent *S. aureus* dissemination and release at other body sites was so far reported, notwithstanding the fundamental role of natural killer cells in the interactions between *S. aureus* and other immune cells of the innate and adaptive immune systems (Small *et al*. 2008; Souza-Fonseca-Guimaraes, Adib-Conquy and Cavaillon 2012).

Altogether, it seems that an ‘appropriate’ interaction of *S. aureus* with blood cells is not only fundamental for the bacterial survival upon invasion, but also for its dissemination throughout the human body. *Staphylococcus aureus* strains have acquired various tools to use the different immune cells as vectors. Whether immune cells will transport *S. aureus* over short or long distances inside the human body will depend on different parameters, including the natural life span of the different types of immune cells, their localization to certain body sites, tissues, the bloodstream or lymphatic vessels, and obviously their survival upon *S. aureus* internalization.

## CONCLUDING REMARKS

Over the past decade, an increasing number of studies have demonstrated the high impact of endogenous *S. aureus* reservoirs on the dissemination of this pathogen through the human body and on the development of infection. Moreover, several studies have advanced our understanding of the interactions of *S. aureus* and various types of blood cells that serve to maintain the homeostasis of the human body upon entry of pathogenic bacteria. Furthermore, much information has been gathered on how the bacteria can escape our immune defenses and even hide within blood cells, leading to an immune imbalance and disease development. Nonetheless, many questions have remained unanswered and further investigations should be performed to better understand the mechanisms underlying the transformation of *S. aureus* from a colonizer into dangerous pathogen. In this respect, our present knowledge of the different *S. aureus* reservoirs in the human body is still very incomplete, especially where it concerns the GI tract. For example, related to identifying the presence of *S. aureus* in the human gut, the precise procedure for its detection is crucial, since the absolute numbers of this bacterium may be low compared with other gut-resident microbes. In addition, *S. aureus* bacteria are quite robust and resilient to the applied lysis protocols, which may introduce a bias in the detection of its DNA through metagenomics approaches. These factors are likely to lead to an underappreciation of *S. aureus* presence in the human gut, and they underpin the need for culture-based quantification of the relative abundance of this bacterium among the gut microbiota in different human populations. Additionally, longitudinal studies should be conducted to investigate, over time, the immune imprint of *S. aureus* during both nasopharynx and GI tract colonization to the subsequent development of infections that emerge from these endogenous reservoirs. To date, most of the available information on *S. aureus* colonization is related to nasal carriage only, or to the presence of *S. aureus* in chronic wounds or the lungs of cystic fibrosis patients. Large-scale systematic studies on other *S. aureus* reservoirs, especially the gut, still need to be carried out. Also, more studies could be conducted over time on known intestinal *S. aureus* carriers to better understand whether intestinal carriage does significantly contribute to the onset of infections as this was previously done for nasal carriers. Studies using intestinal *in vitro* and *ex vivo* models could be conducted to elucidate the mechanisms of *S. aureus* interaction with the different cells present in the GI tract. Additionally, studies on the local immune response after nasal colonization, and on the synergistic effects of the nasal microbiota are also necessary to better understand the interactions between the bacteria in the nasopharynx and the human host. A related question that needs to be explored more in depth concerns the role of blood cells as trojan horses for bacterial dissemination through the human body. Novel sensitive technologies and experimental setups to track infected blood cells are needed to further investigate the extent to which circulating blood cells carry *S. aureus*, especially in relation to nasal and intestinal carriage of this pathogen. In this respect, most hypotheses are focused on neutrophils only, but these immune cells are relatively short-lived. In particular, investigations on the possible roles of other types of blood cells, such as dendritic cells, monocytes, macrophages and natural killer cells, as silent carriers of *S. aureus* will be highly relevant. A better understanding of such mechanisms of *S. aureus* dissemination within the body will be highly relevant for the prevention of postoperative wound infections and infections of prosthetic implants. A clear link between endogenous reservoirs and postoperative wound infection has already been established, but too little is presently known about the routes that *S. aureus* takes from its site of residence to a surgical wound or a prosthetic implant. Lastly, to prevent infections, it will be important to know at which stage the human immune defenses fail, and which of the many *S. aureus* factors implicated in immune evasion are decisive in the fight against infection within the human body. Only then will we be able to fully appreciate the invasive behavior of *S. aureus*, the nature of the unknown triggers that transform the colonizer into the pathogen and the best ways to prevent and treat infections.

## ACKNOWLEDGMENTS

We thank Marines du Teil Espina for stimulating discussions and for help in making the figures, and we thank Marina López-Álvarez for stimulating discussions.

## AUTHORS’ CONTRIBUTION

EJMR drafted the manuscript. DA collected literature and contributed manuscript sections. EJMR and DA prepared the figures. JMVD supervised the project. All authors critically revised the manuscript, gave final approval and agreed to be accountable for all aspects of the review.
